# Transcriptomics identify the triggering of citrate export as the key event caused by manganese deficiency in *Aspergillus niger*

**DOI:** 10.1128/spectrum.01906-24

**Published:** 2024-10-08

**Authors:** Erzsébet Fekete, Vivien Bíró, Alexandra Márton, István Bakondi-Kovács, Erzsébet Sándor, Béla Kovács, Nicholas Geoffrion, Adrian Tsang, Christian P. Kubicek, Levente Karaffa

**Affiliations:** 1 Department of Biochemical Engineering, Faculty of Science and Technology, University of Debrecen, Debrecen, Hungary; 2 University of Debrecen, Juhász-Nagy Pál Doctoral School of Biology and Environmental Sciences, Debrecen, Hungary; 3 Institute of Food Science, Faculty of Agricultural and Food Science and Environmental Management, University of Debrecen, Debrecen, Hungary; 4 Centre for Structural and Functional Genomics, Concordia University, Montreal, Québec, Canada; 5 Institute of Chemical, Environmental and Bioscience Engineering, TU Wien, Vienna, Austria; University of Wisconsin-Madison, Madison, Wisconsin, USA

**Keywords:** *Aspergillus niger*, citric acid, manganese ions, citrate exporter, *cexA*, transcriptomics, gene expression

## Abstract

For over a century, the filamentous Ascomycete fungus *Aspergillus niger* has played a pivotal role in the industrial production of citric acid. A critical fermentation parameter that sustains high-yield citric acid accumulation is the suboptimal concentration of manganese(II) ions in the culture broth at the early stages of the process. However, the requirement for this deficiency has not been investigated on a functional genomics level. In this study, we compared the transcriptome of the citric acid hyper-producer *A. niger* NRRL2270 strain grown under citric acid-producing conditions in 6-L scale bioreactors at Mn^2+^ ion-deficient (5 ppb) and Mn^2+^ ion-sufficient (100 ppb) conditions at three early time points of cultivation. Of the 11,846 genes in the genome, 963 genes (8.1% of the total) were identified as significantly differentially expressed under these conditions. Disproportionately high number of differentially regulated genes encode predicted extracellular and membrane proteins. The most abundant gene group that was upregulated in Mn^2+^ ion deficiency condition encodes enzymes acting on polysaccharides. In contrast, six clusters of genes encoding secondary metabolites showed downregulation under manganese deficiency. Mn^2+^ deficiency also triggers upregulation of the *cexA* gene, which encodes the citrate exporter. We provide functional evidence that the upregulation of *cexA* is caused by the intracellular accumulation of citrate or acetyl-CoA and is a major factor in triggering citrate overflow.

**IMPORTANCE:**

Citric acid is produced on industrial scale by batch fermentation of the filamentous fungus *Aspergillus niger*. High-yield citric acid production requires a low (<5 ppb) manganese(II) ion concentration in the culture broth. However, the requirement for this deficiency has not been investigated on a functional genomics level. Here, we compared the transcriptome of a citric acid hyper-producer *A. niger* strain grown under citric acid-producing conditions in 6-L scale bioreactors at Mn^2+^ ion-deficient (5 ppb) and Mn^2+^ ion-sufficient (100 ppb) conditions at three early time points of cultivation. We observed that Mn^2+^ deficiency triggers an upregulation of the citrate exporter gene cexA and provides functional evidence that this event is responsible for citrate overflow. In addition to the industrial relevance, this is the first study that examined the role of Mn^2+^ ion deficiency in a heterotrophic eukaryotic cell on a genome-wide scale.

## INTRODUCTION

Selected strains of the fungus *Aspergillus niger* are capable of converting up to 90% of 20% (wt/vol) glucose or sucrose to citric acid. This is currently the primary means of industrial citric acid production (1). However, successful performance of this fermentation in submerged mode depends on an interplay of several environmental factors and nutrients, most notably the concentration of Mn^2+^ ions in the medium (2). The high yields of citrate are only reached when the Mn^2+^ concentration in the culture medium is well below 10 ppb (3). The critical role of Mn^2+^ has recently been demonstrated by the deletion of *dmtA*, the gene encoding an NRAMP-family Mn^2+^ transporter, which results in the accumulation of high amounts of citric acid in the presence of Mn^2+^ (4). Besides the consequences for citric acid accumulation, the biochemical and physiological effects caused by this Mn^2+^ deficiency include drastic changes in hyphal morphology (5), chemical composition of the cell wall (5) and the plasma membrane (6), and elevated intracellular protein degradation (7). The role of these changes in citric acid accumulation has been investigated on a functional genomics level. Dai et al. (8) used suppression subtractive hybridization and identified 22 genes that respond to the availability of Mn^2+^ ions. The 10 genes that they identified with deduced functions are involved in amino acid metabolism, protein catabolism, or cell regulatory processes. In accordance with the protein degradation cited above (7), the transcripts encoding a pepsin-type protease and polyubiquitin are upregulated at low (10 ppb) Mn^2+^. More recently, Yin et al. (9) performed a transcriptomic analysis of an industrial *A. niger* strain during citrate production and observed differential accumulation of transcripts predicted to be involved in primary metabolism between the growth stage and the production stage.

We previously showed that the effect of Mn^2+^ on citrate production is confined to the early phase of fermentation (10). To gain insights into the mechanism of how Mn^2+^ deficiency makes citric acid accumulate to high levels, we compared the transcriptome of the citric acid producer *A. niger* NRRL2270 at three time points during the onset of citric acid production at Mn^2+^ ion-deficient (5 ppb) and Mn^2+^ ion-sufficient (100 ppb) fermentation conditions. We observed that Mn^2+^ deficiency triggers an upregulation of the citrate exporter gene *cexA* and provides functional evidence that this event is caused by intracellular accumulation of citrate or acetyl-CoA and is responsible for citrate overflow.

## RESULTS

### Time points for transcriptome analysis

The effect of manganese ions on citric acid accumulation by *A. niger* is dependent on the cultivation time (10): it prevents citric acid accumulation most strongly when added at the onset of the cultivation, but the effect is much weaker when added at a later stage of growth. Thus, the addition of 100 ppb Mn^2+^ at the beginning of the fermentation reduces molar citric acid yield (*Y*_*p*/*s*_) to 0.35 (compared to 0.73 in the presence of only 5 ppb Mn^2+^), whereas the addition of 100 ppb after 72 hours of cultivation leads to an *Y*_*p*/*s*_ of 0.71 and therefore hardly affects citric acid accumulation (10). Consequently, we compared the transcriptome of *A. niger*, grown under citric acid-producing conditions, in the presence of 5 ppb (= manganese deficiency) and 100 ppb (= manganese sufficiency) Mn^2+^ ions, respectively, at three time points of cultivation: 24 hours, when citric acid just starts to accumulate; 48 hours, when phosphate is already exhausted; and 72 hours, where citric acid accumulation has reached the maximal production rate (for the entire data set, see Table S1).

### Properties of the Mn^2+^-dependent transcriptome

Table S2 shows the expression levels and fold-change of all transcripts under Mn^2+^-deficient and Mn^2+^-sufficient conditions. Using the cut-off criteria described in Materials and Methods, 963 genes were identified whose transcript had a mean TPM value higher than 10 and whose transcription differed between manganese deficiency and manganese sufficiency by a log2 value >2 or <−2 at *P* < 0.05. Since the *A. niger* NRRL3 genome lists 11,846 genes, this means that 8.1% of them are differentially expressed under the present conditions.

**Fig 1:**
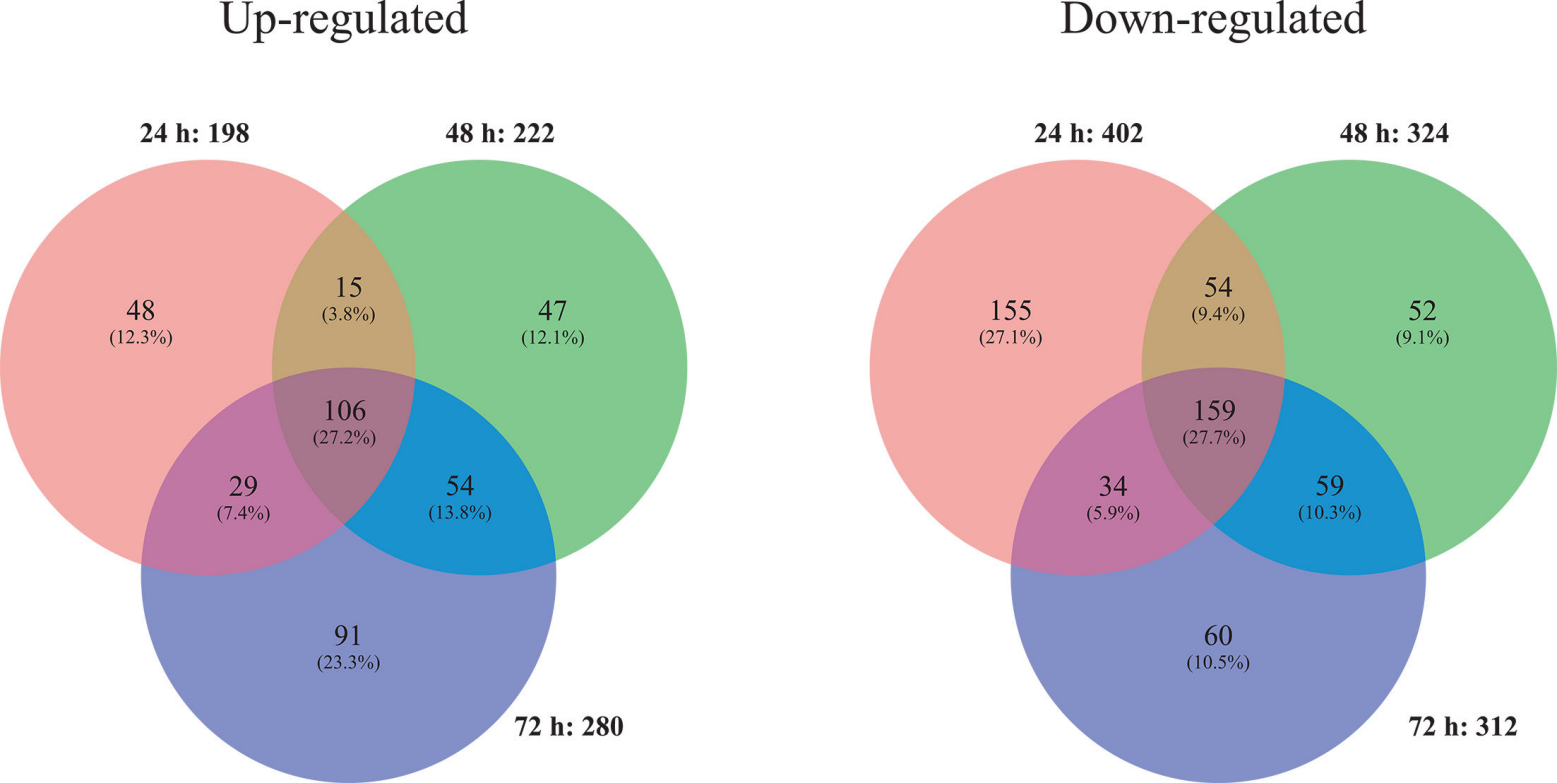
Properties of the manganese(II) ion-dependent transcriptome in the *A. niger* strain NRRL2270, illustrated in a Venn diagram (11). Occurrence of genes that are either up- or downregulated under manganese deficiency is shown at three different time points.

Among the 963 genes, 390 were upregulated and 573 downregulated under manganese deficiency. Figure 1 shows the occurrence of these genes at the three different time points: 106 and 159 genes were up- and downregulated, respectively, under manganese deficiency at all three time points; 48 and 155 transcripts, respectively, were up- or downregulated only after 24 hours. In addition, a minor number of genes were shared by two time points. The up- and downregulation in response to manganese deficiency were very strict: no gene was found that was upregulated at one time point and downregulated at another time point.

The *A. niger* NRRL3 genome contains 2,132 hypothetical proteins (= proteins for which no function can be predicted), which make up for 18% of the total genome. If this number is related to the number of differentially regulated genes (963), one would expect that our transcriptome should include about 173 genes encoding hypothetical proteins. However, a total of 309 hypothetical proteins were encountered. A slightly higher number of them was observed to be upregulated under manganese-deficient conditions (38%, 149 upregulated transcripts vs 28%, 160 downregulated transcripts). Interestingly, in total, 168 predicted and 162 hypothetical proteins were identified to be either secreted, bound to the plasma membrane, or formed transmembrane helices (Table S2), which makes up 17.4% and 16.8 % of the 963 differentially expressed genes. The number of these secreted proteins (predicted and hypothetical) was almost double under manganese deficiency than under manganese sufficiency (38.7% vs 21.3% of the total number of downregulated and upregulated genes, respectively; Table 1).

**TABLE 1:** Predicted location of the proteins encoded by the manganese-dependent transcriptome

Total genes	Mn deficiency		Mn sufficiency	
	390		573	
	Function predicted	Hypothetical proteins	Function predicted	Hypothetical proteins
	241	149	413	160
Predicted cellular location				
Extracellular	92 (23.6 %)	59 (39.6 %)	76 (13.2 %)	73 (28.7 %)
Signal peptide	65	29	24	23
Transmembrane domain(s)	22	25	41	46
Signal peptide and transmembrane domains	5	5	11	4
Intracellular	149	60	337	110

### Characteristics of the manganese-deficient and -sufficient transcriptomes

We manually screened the genes that encoded proteins with a predictable function. The most abundant gene group that was upregulated by manganese deficiency was that encoding CAZymes, enzymes acting on polysaccharides (50 transcripts; Table 2; Table S3). They predominantly encoded enzymes acting on plant biomass polymers [glycosyl hydrolases, auxiliary activity family 9 (AA9)-lytic polysaccharide monooxygenases, polysaccharide lyases (PL), and carbohydrate esterases (CE)].

**TABLE 2:** Major gene groups found in this study and their response to manganese deficiency

CAZymes	AA9-lytic polysaccharide monooxygenase	5	0
	Carbohydrate-binding module	1	1
	CE	4	1
	Glycoside hydrolase	36	9
	Glycosyltransferase	2	12
	PL	1	0
Transport	Amino acid permeases	4	4
	Major facilitator superfamily transporter	13	28
	ATP-binding cassette transporters	1	4
Enzymes	Proteinases	13	3
	Acid phosphatases	0	4
	α/β-Hydrolases	1	18
	Cytochrome P450 monooxygenases	8	12
	Short-chain dehydrogenases/reductases	6	13
Others	Proteins with ankyrin repeats	1	9
	Zn2Cys6 transcriptional regulators	5	7
	Protein kinases	1	6
	Histidine phosphatases	1	6
	Cell wall synthesis	3	6
Secondary metabolism	Non-ribosomal peptide synthetases	0	5
	Polyketide synthases	0	1

In addition to the CAZymes, an increased number of transcripts encoding proteases were encountered under manganese deficiency (Table 2). Most of them encoded aspartic proteases, which can be explained by the acidic pH (below 2) persisting under our cultivation conditions, and this would also be in agreement with data of Dai et al. (8) who identified enhanced transcription of a pepsin-type protease under manganese deficiency by subtractive hybridization. Unfortunately, no accession number was quoted for this gene, and we are therefore unable to tell whether it is identical to the ones we found.

Genes whose transcription was downregulated by manganese deficiency spanned a broad functional range: they encoded proteins with an ankyrin domain, proteins belonging to the α/β-hydrolase superfamily, acid phosphatases, protein kinases, histidine phosphatases, secondary metabolite synthases, and GT (glycosyltransferases) (Table 2). The other major gene groups appeared in comparable numbers under manganese deficiency and sufficiency. We found 12 Zn_2_Cys_6_-trancriptional regulators, of which five were upregulated under manganese deficiency. None of them has been characterized so far, and their genomic location did not provide an insight into their possible function.

The secondary metabolite synthases comprised five non-ribosomal peptide synthases (NRPS) and a single polyketide synthase (PKS). Only one of them had already been characterized before (NRRL3_08538, which encodes the NRPS involved in siderophore biosynthesis). The other five genes are involved in the synthesis of thus far unknown secondary metabolites. Since NRPS- and PKS-encoding genes are usually located in the immediate vicinity of the genes for processing and modification of the respective secondary metabolite, we also looked whether other genes in these six secondary metabolite clusters show the same downregulation under manganese deficiency. As shown in Table S4, this was in part the case. Several genes of the respective clusters were also significantly downregulated. The others showed only a low response, but we note that they were generally very weakly expressed.

We also noted that a higher number of genes involved in cell wall synthesis were downregulated by manganese deficiency (Table 2). Among them, three genes (NRRL3_04198, NRRL3_10495, and NRRL3_09919) encoded putative *KRE9*/*KNH1* homologs. In *S. cerevisiae,* they encode cell surface O-glycoproteins that are required for β-1,6-glucan synthesis in *S. cerevisiae* (12). Their loss of function leads to a dramatically increased chitin content in the cell wall (13). Proteins of the KRE9/KNH1 have not yet been studied in *Aspergillus* spp., but their downregulation under manganese deficiency matches the enhanced cell wall chitin content under these conditions (5).

We also looked for genes involved in metabolism, metal ion transport, and solute transport (Table 3), but no consistent conclusions could be drawn: manganese deficiency strongly stimulates the expression of the mitochondrial aconitase but has no effect on the expression of other genes involved in the tricarboxylic acid cycle. However, expression of an acetyl-CoA hydrolase and an acetate transporter is stimulated, which could be interpreted as an attempt to maintain acetyl-CoA homeostasis. Expression of glutamine-fructose-6-phosphate aminotransferase, an enzyme involved in chitin biosynthesis, was also stimulated by manganese deficiency, which again (see above) coincides with the strongly enhanced chitin content in the cell walls (5). Two genes encoding ferric reductases were also upregulated under manganese deficiency, but the iron siderophore transporters were downregulated (see above) as were also transporters for zinc, magnesium, and chromium ions. This illustrates that manganese deficiency influences the transport of inorganic ions, but the reason for it is unclear.

**TABLE 3:** Genes involved in metabolism, solute transport, and inorganic ion homeostasis that are significantly regulated by manganese deficiency

Metabolism	Solute transport	Inorganic ion homeostasis
**Upregulated under manganese deficiency**		
NRRL3_06777 acetyl-CoA hydrolase	NRRL3_07471 acetate transporter, putative	NRRL3_03124 Ctr copper transporter family protein
NRRL3_08639 acetylglutamate kinase-like protein	NRRL3_08393 amino acid transporter	NRRL3_00668 ferric chelate reductase
NRRL3_00316 aconitate hydratase-mitochondrial	NRRL3_08744 amino acid/polyamine transporter I family protein	NRRL3_06306 ferric reductase domain-containing protein
NRRL3_08137 alpha-hydroxy acid dehydrogenase family protein-FMN dependent	NRRL3_08764 amino acid/polyamine transporter I family protein	NRRL3_03932 Slc26A/SulP transporter family protein
NRRL3_05997 alpha-isopropylmalate isomerase	NRRL3_07694 amino acid/polyamine transporter I family protein	NRRL3_01975 Slc26A/SulP transporter family protein
NRRL3_08469 dihydroxy-acid dehydratase	NRRL3_06530 CexA citrate exporter	
NRRL3_08347 glutamine-fructose-6-phosphate aminotransferase	NRRL3_09134 malate permease	
NRRL3_11179 nitrite reductase	NRRL3_07401 dicarboxylate:proton symporter	
NRRL3_00669 tryptophan synthase subunit beta	NRRL3_06352 purine nucleoside permease	
NRRL3_07971 type I mannose-6-phosphate isomerase-like protein		
NRRL3_07229 UDP-galactopyranose mutase—involved in cell wall biosynthesis		
**Downregulated under manganese deficiency**		
NRRL3_02437 UDP-glucose 6-dehydrogenase	NRRL3_00402 choline permease	NRRL3_01725 chromate transporter
NRRL3_02448 2-methylcitrate dehydratase	NRRL3_02722 amino acid/polyamine transporter I family protein	NRRL3_02168 low-affinity zinc transporter-plasma membrane
NRRL3_02449 citrate synthase-like protein	NRRL3_04377 purine permease	NRRL3_03426 zinc/iron permease family protein
NRRL3_02450 *cis*-aconitate decarboxylase	NRRL3_10037 amino acid permease/SLC12A domain-containing protein	NRRL3_06368 magnesium transporter CorA/zinc transporter ZntB family protein
NRRL3_02523 L-arabinitol 4-dehydrogenase LadA		NRRL3_07020 vacuolar ion transporter
NRRL3_06150 isochorismatase-like domain-containing protein		NRRL3_07300 calcium permeable stress-gated cation channel 1
NRRL3_06201 ketopantoate reductase ApbA/PANE-like protein		NRRL3_10131 metalloreductase
NRRL3_06930 D-galacturonate reductase		
NRRL3_07437 3-isopropylmalate dehydrogenase Leu2B		
NRRL3_07585 orotate phosphoribosyltransferase		
NRRL3_07594 phosphoenolpyruvate synthase 2-like protein		
NRRL3_07882 phenylalanine ammonia-lyase		
NRRL3_08606 mannitol 2-dehydrogenase		
NRRL3_08612 D-serine dehydratase-like protein		
NRRL3_08837 L-rhamnose-1-dehydrogenase		
NRRL3_09244 trehalose 6-phosphate phosphatase		
NRRL3_09935 phosphoenolpyruvate carboxykinase-ATP utilizing		
NRRL3_11035 dehydroshikimate dehydratase		
NRRL3_11144 oleate delta-12 desaturase		

### Overexpression of cexA overcomes the inhibition of citric acid accumulation by manganese ions

**Fig 2:**
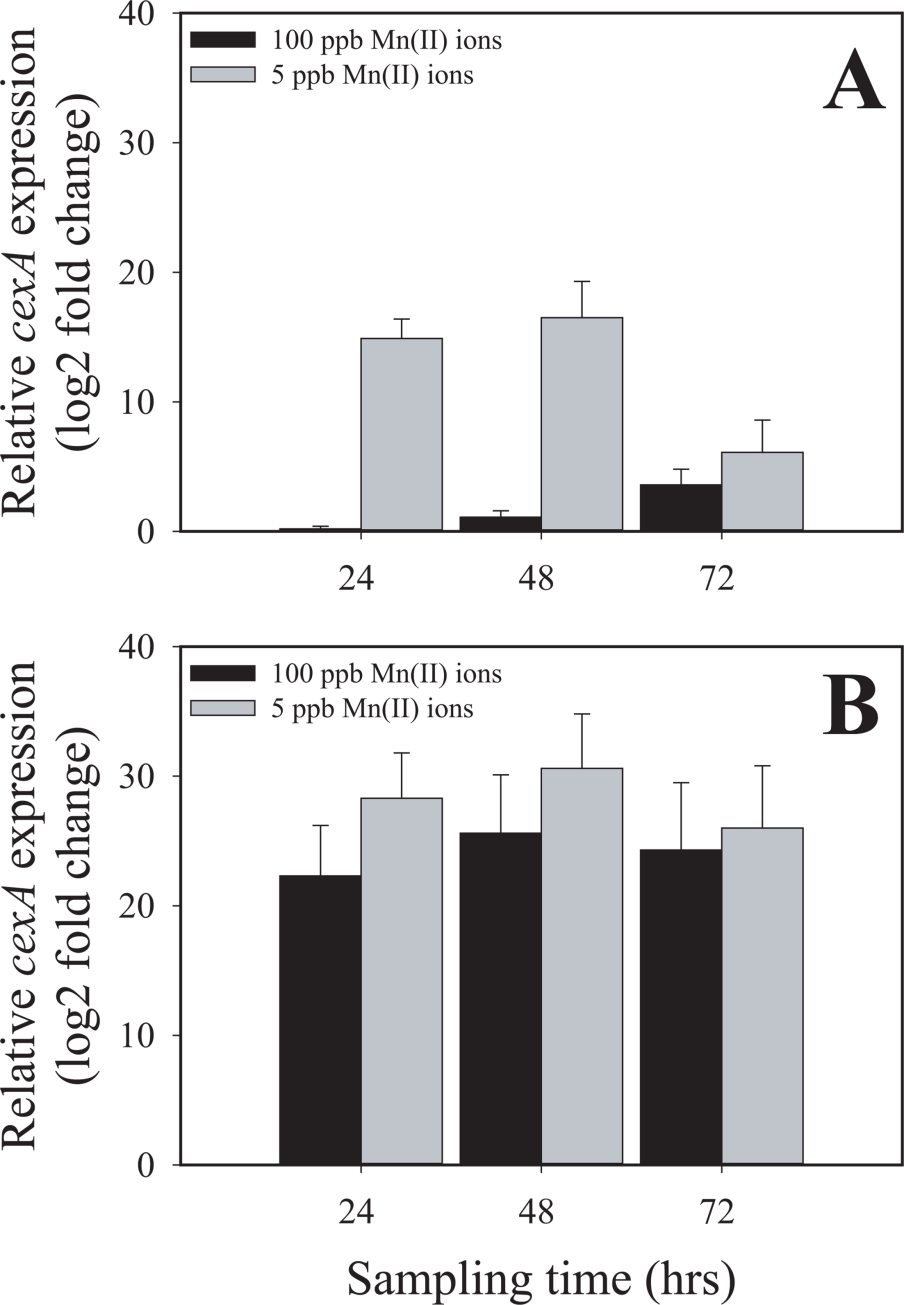
Transcript levels of the citrate exporter *cexA* under Mn-deficient (= 5 ppb) and Mn-sufficient (= 100 ppb) conditions in the *A. niger* NRRL2270 strain (panel A) and the *ΔglaA::cexA*-overexpressing mutant (panel B) at 24, 48, and 72 hours of cultivation. All samples were referenced to the 24-hour, 100-ppb Mn(II) sample. The constitutive (“housekeeping”) *actA* was used as reference gene.

**Fig 3:**
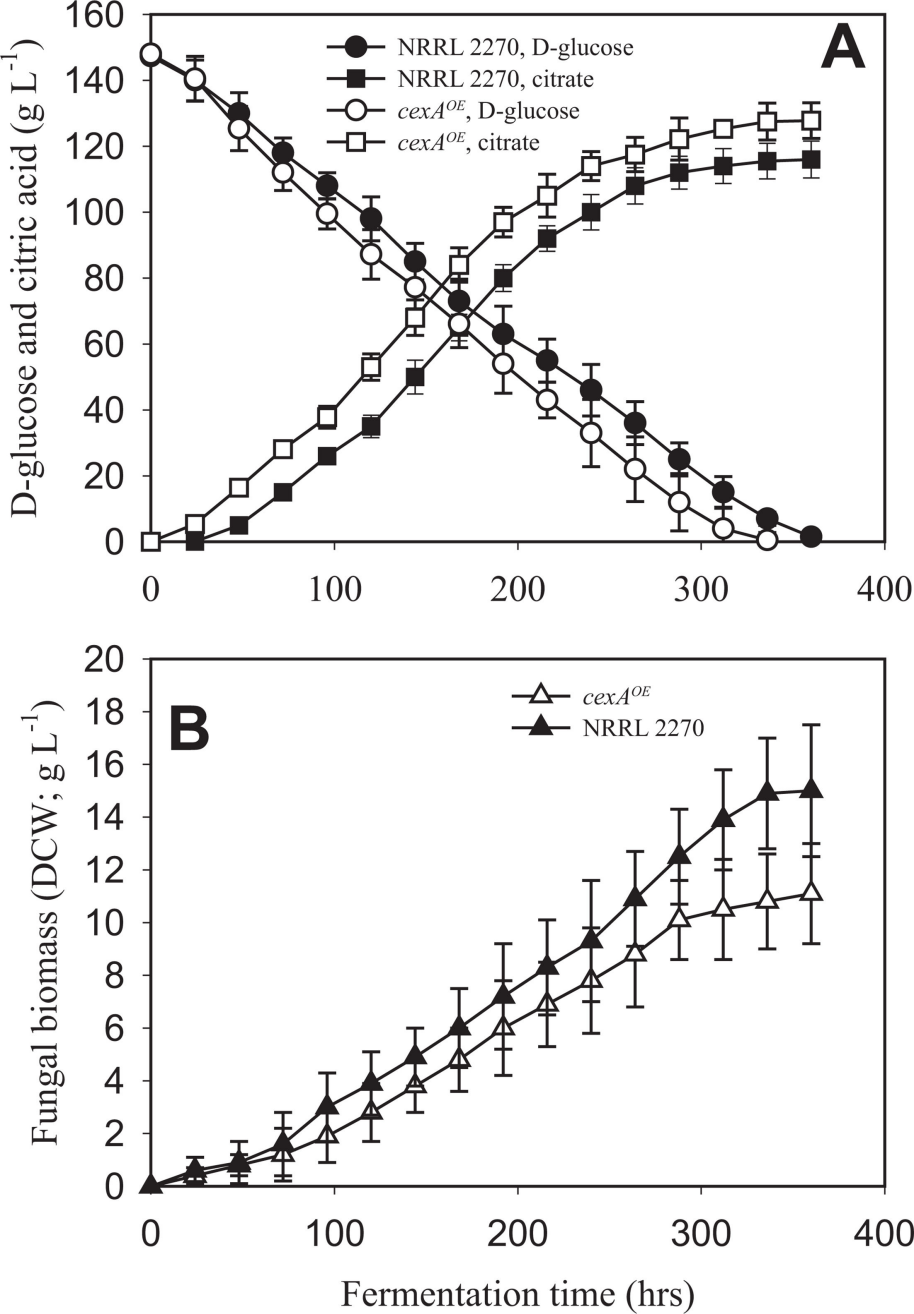
Kinetics of D-glucose utilization and citric acid production (panel A) and biomass formation (panel B) of the *A. niger* NRRL 2270 strain and the *ΔglaA::cexA*-overexpressing mutant, grown under manganese-deficient (= 5 ppb, citrate-producing) conditions. Fermentations were carried out in triplicate. Standard deviations are indicated with vertical bars for each determined value. Note that the bar is sometimes smaller than the symbol that marks the mean concentration. See Materials and Methods for further details.

**Fig 4:**
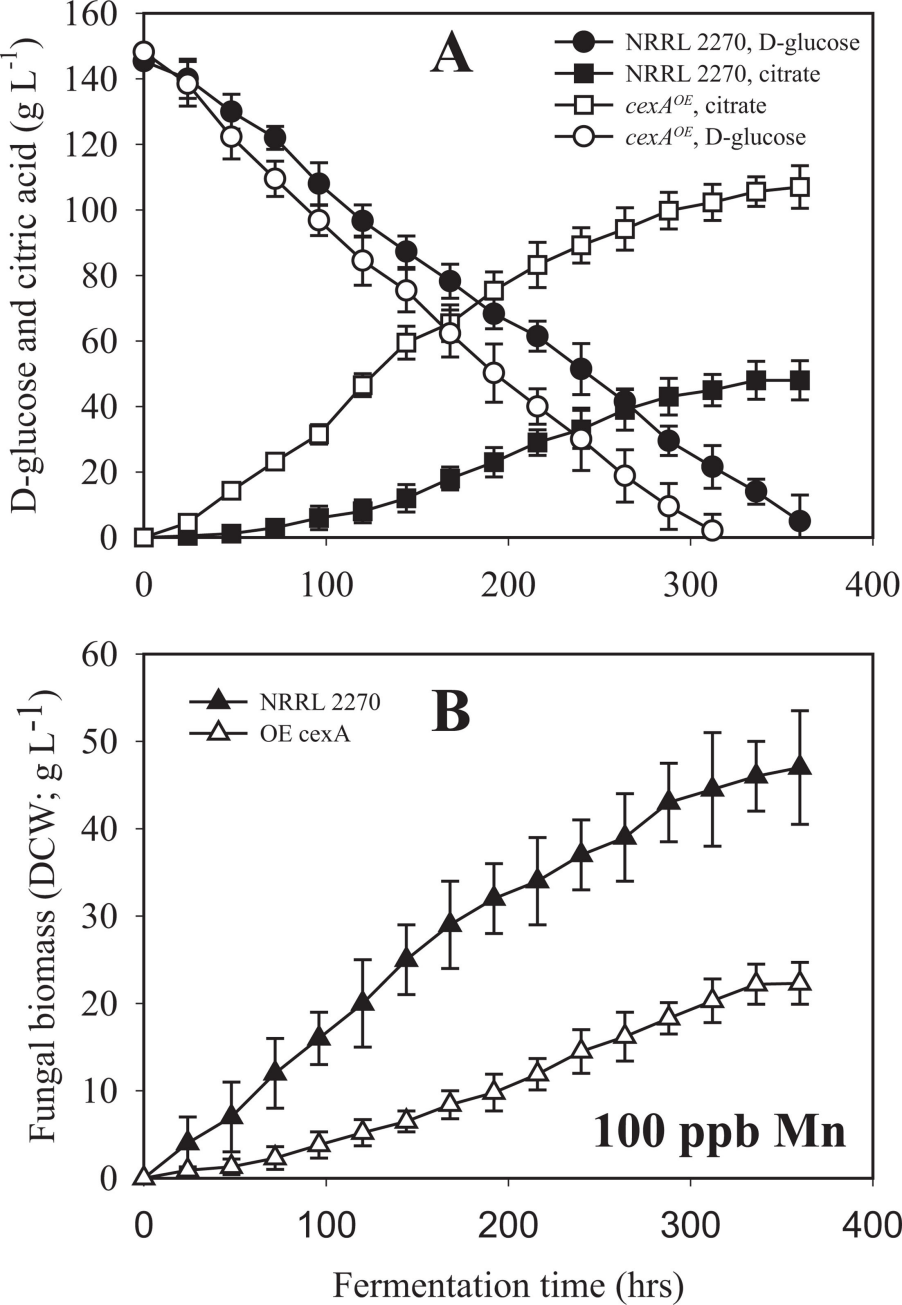
Kinetics of D-glucose utilization and citric acid production (panel A) and biomass formation (panel B) of the *A. niger* NRRL 2270 strain and the *ΔglaA::cexA*-overexpressing mutant, grown under manganese-sufficient (= 100 ppb, citrate-non-producing) conditions. Fermentations were carried out in triplicate. Standard deviations are indicated with vertical bars for each determined value. Note that the bar is sometimes smaller than the symbol that marks the mean concentration. See Materials and Methods for further details. DCW, dry cell weight.

We detected the upregulation of the gene encoding CexA (NRRL3_06530), which is responsible for citrate export (14), at 24 and 72 hours under manganese deficiency. To verify the upregulation of *cexA* under manganese deficiency, we analyzed by quantitative RT-PCR its transcript levels normalized to those of the constitutive *actA* (actin-encoding) gene transcripts (Fig. 2A). This analysis confirmed the very low transcription of *cexA* in the presence of 100 ppb Mn^2+^, particularly at 24 and 48 hours of cultivation, and also the significant *cexA* upregulation at 24 and 48 hours of cultivation under manganese deficiency. This led us to speculate that the stimulation of *cexA* transcription could be a crucial factor in manganese deficiency for high citric acid production. To test this, we replaced the coding region of the *glaA* (glucoamylase) gene with the coding region of *cexA* (Δ*glaA:cexA*) that expresses *cexA* under the maltose/glucose-inducible *glaA* promoter (15). This strain will henceforth be termed *cexA^OE^*. Figure 2B shows that this led to Mn^2+^ -independent high levels of the *cexA* transcript, which surpassed that observed in the parent strain under manganese limitation. Consequently, we cultivated the *A. niger* NRRL2270 strain and *cexA^OE^* in a citric acid-producing medium. Under manganese deficiency, both strains accumulated a high concentration of citric acid. *A. niger cexA^OE^* accumulated a little more than the parent strain (120 vs 110 g·L^−1^), which was due to an earlier start of citrate excretion because the volumetric production rates were similar between the two strains (Fig. 3A and B). In the presence of 100 ppb Mn^2+^ ions, which results in a final citric acid concentration of only 40 g·L^−1^ in the parent strain, *cexA^OE^* produced up to 100 g·L^−1^ citric acid corresponding to 83% of the concentration that accumulated under manganese limitation (Fig. 4A and B). From these data, we conclude that the upregulation of *cexA* under manganese limitation is a major reason why this condition is necessary for high-yield citric acid accumulation.

### Addition of extracellular citric acid triggers cexA expression irrespective of the presence of manganese ions

The mechanism of *cexA* upregulation by manganese limitation is not yet clear. Recently, it was shown (16) that Mn^2+^ ions suppress transcription of *cexA* and decrease the secretion of citric acid in *A. niger*, implying that *cexA* upregulation is in fact a derepression. In order to test this, we grew *A. niger* on a citric acid-producing medium at both manganese deficiency and sufficiency, but with only 1% (wt/vol) glucose. No citric acid is accumulated at this carbon source concentration, independently of the concentration of manganese ions. The transcript of *cexA* was not detected either under manganese deficiency or manganese sufficiency, demonstrating that manganese deficiency does not derepress *cexA* transcription in the presence of low concentrations of glucose.

**Fig 5:**
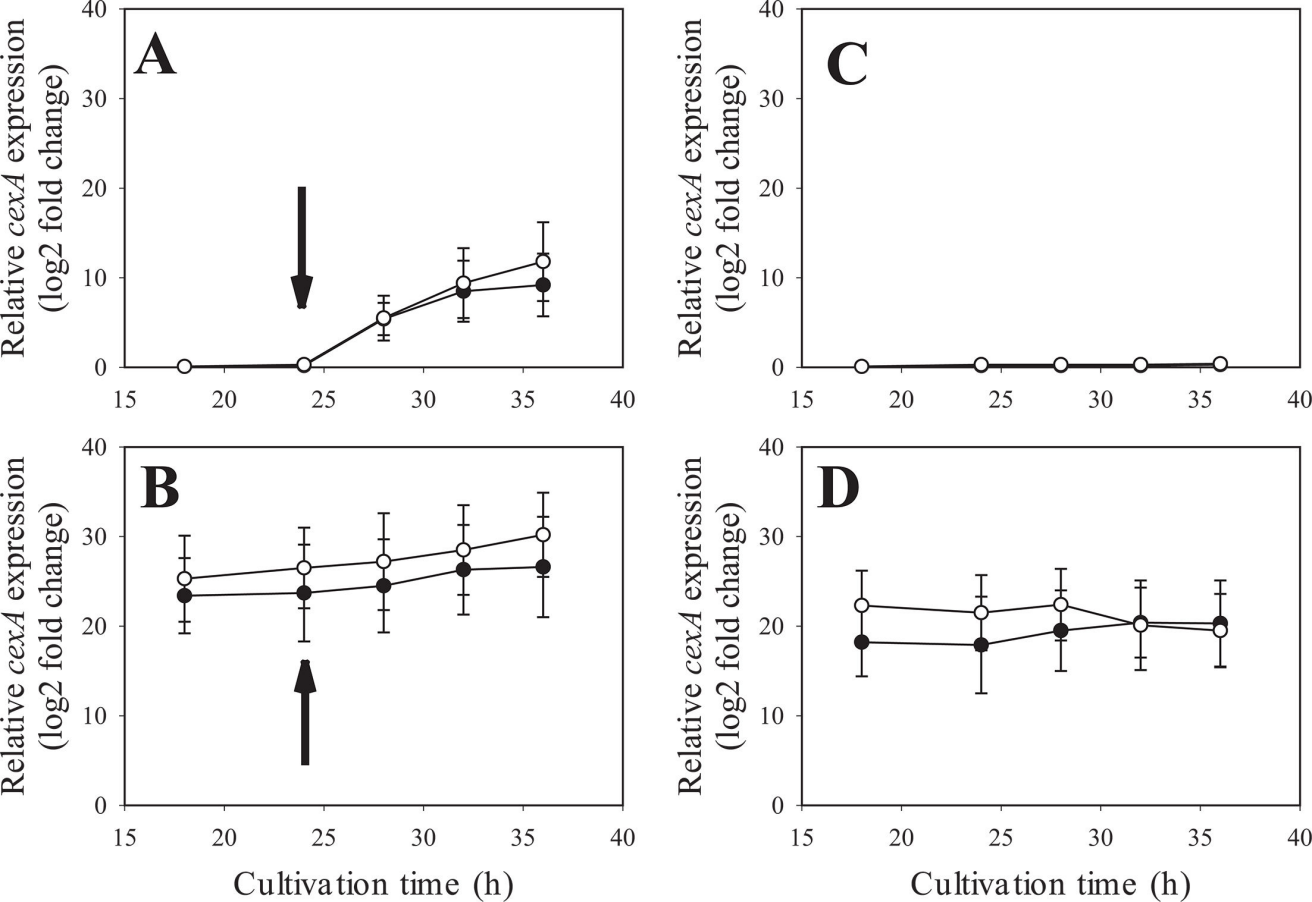
Expression levels of the *cexA* gene in *A. niger* cultures upon external citric acid pulse of 10 g·L^−1^ (indicated by the arrows) as well as in the negative control cultures (no citric acid pulse). Cultures were grown in 500 mL shake flasks under Mn-deficient (= 5 ppb, white symbols) and Mn-sufficient (= 100 ppb, black symbols) conditions. Fermentation parameters were identical to those in the previous experiments, except for the initial D-glucose concentration that was set at 10 g·L^−1^. Panel A: *A. niger* NRRL 2270 strain. Panel B: *ΔglaA::cexA*-overexpressing mutant. Panel C: control culture for the *A. niger* NRRL 2270 strain. Panel D: control culture for the *ΔglaA::cexA*-overexpressing mutant.

We posit that the metabolism at the onset of growth in a manganese-limited citric acid fermentation medium creates a metabolite, which induces *cexA*. We surmized that this could be citrate itself or a metabolite of its biosynthesis. To test this, we again grew *A. niger* on 1% glucose as before and pulsed the culture after 24 hours with citric acid (10 g·L^−1^). This led to the expression of *cexA* independent of manganese ion deficiency or sufficiency (Fig. 5). We conclude that manganese ions are not repressors of *cexA* transcription, but its upregulation is triggered by the accumulation of citric acid or a metabolite related to its metabolism.

## DISCUSSION

This is the first study that examined the role of manganese ion deficiency in a heterotrophic eukaryotic cell on a genome-wide scale. When interpreting the data, however, one needs to be cautious because the medium in which the fungus was grown is unusual [high-carbohydrate concentration, low pH, and high dissolved oxygen (DO) tension]. Many effects caused by manganese deficiency may have become masked by the composition of the medium. As an example, the manganese-dependent superoxide dismutase (NRRL_02664) was slightly downregulated (log2-fold = −1.3, *P* = 1.4E−10) and did not pass the criteria used in our transcriptome analysis. Likewise, adenylate cyclase (NRRL3_10022) was only weakly enhanced (log2-fold = 0.76, *P* = 1.3E−35). Our data are therefore only valid under conditions used for citric acid production at high yields and should not be used for the interpretation of physiology that develops under other cultivation regimes.

The most striking group of genes that were upregulated under conditions of manganese ion limitation was those encoding various CAZymes, including cellulases, hemicellulases, and pectinases. This was particularly surprising because cultivation was done on D-glucose as the only carbon source, and expression of these genes has been described to be dependent on various inducers (17). We also checked the expression of the three characterized transcriptional activators of cellulase and hemicellulose gene expression [XlnR, NRRL3_4034; ClrA, NRRL3_3544; and ClrB, NRRL3_9050; (16)], but it did not react to the presence or absence of manganese ions in the medium. However, this finding is not without precedent: similar set of CAZyme-encoding genes were shown to be downregulated in an *A. niger* mutant (*scl-2*), in which the repressor of cleistothecium formation was impaired (18). Like in our study, no inducers for CAZyme gene expression were added. This implies that the expression of CAZymes in *A. niger* may respond to developmental signals. In the Sordariomycete *Trichoderma reesei*, plant polysaccharide-degrading CAZymes are upregulated during conidiation (19). A small number of CAZyme-encoding genes—particularly GT—were, in contrast, downregulated, and they encompassed mainly enzymes that are supposed to be involved in cell wall biosynthesis (Table S3). Together with the downregulation of three KRE9/KNH1 family β-1,6-glucan synthases, this illustrates a significant difference in cell wall metabolism under manganese deficiency, which is likely also the reason for the differences in hyphal morphology (5).

We also identified five NRPS and one PKS that were strongly downregulated under manganese deficiency, and we also showed that several other genes in the biosynthesis clusters of these secondary metabolite synthetases behave the same. Although *Aspergillus* spp. are frequently used as model organisms for studying the regulation of secondary metabolism, a role of Mn^2+^ in their biosynthesis has not yet been investigated. However, in *Penicillium urticae*, Mn^2+^ ions have been shown to be necessary for patulin biosynthesis (20). The molecular mechanism has not been elucidated, but inhibitor studies showed that manganese exercised its effect by influencing the coordinated transcription of the enzymes for biosynthesis of patulin (21). This is in agreement with our results that several PKS and NRPS genes of *A. niger* are only transcribed in the presence of Mn^2+^ ions. Most of these genes have unfortunately not yet been characterized, and it is therefore not possible to interpret a reason for this observation.

A major finding of this study is that *cexA*, which encodes the citrate exporter, is upregulated under manganese ion limitation. Placing the *cexA* gene under the strong *glaA* promoter resulted in a strain that produces citric acid under conditions of manganese sufficiency of up to 83% of the wild-type strain under manganese deficiency. This illustrates that *cexA* upregulation is a major—if not the dominant—reason why citric acid production requires manganese deficiency. Reinfurt et al. (16) obtained similar results with *A. niger* ATCC 1015, and the effect was interpreted as a repression of *cexA* by manganese ions. However, no *cexA* expression was detected under manganese deficiency when growing on a medium with only 1% (wt/vol) glucose, and the transcriptome of cells grown under manganese sufficiency did not reveal any DNA-binding protein that could act as such a repressor.

The protein S-methyltransferase LaeA has been identified to be essential for citric acid accumulation in *A. niger* (22), *A. carbonarius* (23), *A. oryzae* (24), and *Aspergillus luchuensis* mut. *kawachii* (25). The latter authors also identified that LaeA was essential for the expression of *cexA* via acting on methylation levels of the histones H3K4 and H3K9, and overexpression of *cexA* in a Δ*laeA* mutant strain rescued citric acid production. They concluded that the necessity of citric acid accumulation for functional LaeA is due to its requirement for the expression of *cexA*. Reinfurt et al. (16) speculated that the absence of manganese ions may lead to LaeA activation that in turn stimulates *cexA* expression. However, this study demonstrates that LaeA (NRRL3_02676) is strongly downregulated under manganese deficiency and only upregulated in the presence of manganese ions. The induction of *cexA* expression by manganese deficiency can therefore not involve LaeA. The reason for this difference to the earlier studies is unclear, but we would like to note that all the above cited studies on the effect of LaeA on citric acid production have been performed without the removal of manganese ions from the medium and thus resulted only in very low citric acid concentrations (40, 8, and 10 g/L (21, 22, 24), respectively). It would be interesting to know whether the effect of LaeA indeed occurs under high-yielding conditions at all.

Our data rather point to the inability to trigger the expression of *cexA* in the presence of 100 ppb manganese ions. What could be such a trigger? The role of CexA is to export citrate from the cytoplasm to the extracellular medium. But cytosolic citrate is the precursor for acetyl-CoA used in fatty acid biosynthesis, and citrate should therefore only be exported when it exceeds a critical concentration that would disturb this process. It is therefore reasonable to assume that triggering of *cexA* transcription will only occur then. These theoretical considerations are supported by the fact that the first increase of citric acid concentration in the early phase of citric acid fermentation occurs only intracellularly (26), and its first extracellular detection occurs about 10 hours later when the intracellular concentration is already at 9 mM. We therefore sought for a method to manipulate the intracellular citric acid concentration on 1% glucose medium. One possibility would have been to knock out either *acl1* or *acl2,* the genes encoding the two subunits of ATP-citrate lyase. However, such strains have been reported to accumulate succinic acid (27) and have several developmental defects (28). Since citric acid is known to be taken up by *A. niger* growing on low-glucose concentrations (29), we pulsed 1% (wt/vol) glucose cultures with citric acid in the presence of 5 and 100 ppb manganese ions. Indeed, *cexA* expression was triggered by this treatment, and similar transcript levels were obtained under manganese deficiency and sufficiency, respectively. Performing the same citrate-pulsing experiment with the *cexA*-overexpressing strain had no effect on *cexA* expression, indicating that the effect is specific for the *cexA* promoter. Whether the triggering metabolite is indeed citric acid or acetyl-CoA (or another metabolite formed from citrate) cannot be predicted at this time. Liu et al. (30) showed that the generation of an enhanced supply of cytosolic acetyl-CoA by introducing a bacterial phosphoketolase pathway into *A. niger* significantly enhances citric acid production. The authors interpreted their findings by assuming that the cytosolic citrate synthase CitB receives an increased concentration of its substrate acetyl-CoA and therefore enhances citric acid synthesis. However, under the present conditions, only the mitochondrial citrate synthase CitA (NRRL3_00547) was significantly expressed, whereas the other three citrate synthases (NRRL3_00288, NRRL3_02449, CitB NRRL3_11764) together conferred less than 1% of the expression of CitA. An increase in citrate accumulation by enhanced substrate supply for CitB under our conditions is therefore unlikely. Yet the demonstration that an increased concentration of acetyl-CoA stimulates citric acid accumulation suggests that the *cexA* expression triggering metabolite could indeed be acetyl-CoA. Our findings of a stimulated expression of acetyl-CoA hydrolase and acetate transport under manganese deficiency suggest that the acetyl-CoA concentration is likely enhanced under this condition.

This study revealed that most genes for energy and carbon metabolism remained unaffected by manganese deficiency. This contrasts with the findings of Yin et al. (9) who examined the transcriptome of an industrial *A. niger* citrate producer under citric acid production conditions. They report the upregulation of many genes of glycolysis and the TCA cycle. While their study and ours are not directly comparable because they investigated the transcriptome only from two time points of the same strain while we compared the effect of manganese ions, there is also another major difference: they used a cut-off of twofold, whereas we used log2-fold (i.e., the double cut-off). Our data support the view that citric acid accumulates through activation and inhibition by certain metabolites of the glycolysis and the TCA cycle rather than by stimulation of the expression of the pathway genes (31).

## MATERIALS AND METHODS

### *Aspergillus niger* strains, media, and cultivation conditions

*Aspergillus niger* NRRL2270 (A60; ATCC 11414), a hyper-producing strain (2, 32), was maintained at 4°C as conidiospores on agar plates containing minimal medium (pH 6): 10 g D-glucose L^−1^, 6 g NaNO_3_ L^−1^, 1.5 g KH_2_PO_4_ L^−1^, 0.5 g MgSO_4_·7 H_2_O L^−1^, and 0.5 g KCl L^−1^, supplemented with 20 µL trace element solution [containing per liter: 10 g EDTA, 4.4 g ZnSO_4_·7 H_2_O, 1.01 g MnCl_2_·4 H_2_O, 0.32 g CoCl_2_·6 H_2_O, 0.315 g CuSO_4_·5 H_2_O, 0.22 g (NH_4_)_6_Mo_7_O_24_·4 H_2_O, 1.47 g CaCl_2_·7 H_2_O, 1.1 g FeSO_4_·7H_2_O].

Seed cultures were inoculated with 5 × 10^6^ *A. niger* conidia per mL of growth medium from a freshly prepared, high-density spore suspension in a 0.01% Tween 20 solution. Seed cultures were grown for 24 hours in 500 mL Erlenmeyer (conical) flasks (VWR International Kft., Debrecen, Hungary) containing 100 mL of media in a rotary shaker (Infors AG, Basel, Switzerland) operating at 250 rpm at 30°C. Seed culture medium contained D-glucose as a sole carbon source at an initial level of 10 g·L^−1^ and additionally contained 2.50 g (NH_4_)_2_SO_4_, 0.15 g KH_2_PO_4_, 0.15 g NaCl, 2.25 g MgSO_4_·7H_2_O, 1.50 mg Zn^2+^, 0.10 mg Fe^2+^, and 0.06 mg Cu^2+^ per liter. The initial medium pH was set at 3.0 with 3 M HCl and was not controlled during the shake flask cultivations.

Production cultures were grown in a chemically defined medium identical to the seed culture medium except that the initial D-glucose concentration was set at 140 g·L^−1^. To control the concentration of Mn^2+^ in the growth medium, D-glucose was dissolved in distilled water and passed through a column (440 × 45 mm) of Dowex 50 W-X8 (100/200) cation exchange resin. All components were added to this D-glucose solution from sterile stock solutions. The final Mn^2+^ concentration was adjusted with MnCl_2_·4 H_2_O to 5 and 100 µg·L^−1^ for manganese-deficient and -sufficient (= control) cultures, respectively. The growth media thus prepared were membrane filtered under aseptic conditions into the heat-sterilized and cooled shake flasks or bioreactors (fermenters), containing the necessary volume of ion-exchanged and, subsequently, double-distilled water (henceforth referred to as Mn^2+^-free water). All chemicals used were of analytical grade and purchased from Sigma-Aldrich Ltd. (Budapest, Hungary).

Citric acid fermentations were carried out in batch mode in a pair of 6-L-scale glass bioreactors (Sartorius Biostat B, Göttingen, Germany). Prior to this study, all their stainless steel components (headplate, sensor housings, agitator shaft, sampling tube, impellers) were subjected to electrochemical polishing (carried out by Zolend Ltd., Debrecen, Hungary) to prevent excessive manganese leaching (10). The bioreactors were autoclaved in empty vessel mode, i.e., the vessel contained just a minimal amount of Mn^2+^-free water to cover the tips of the pH- and DO sensors. Following cooling, water was forced out by overpressure, and the vessels were filled to the required levels with sterilized growth medium under aseptic conditions. This way, manganese leaching from the metal parts of the bioreactor that inevitably occurs during sterilization was minimized (10).

Operating conditions were 30°C and 0.75 vessel volume per minute of aeration. The initial pH of the growth medium was adjusted to 3.0 with 3 M HCl before inoculation. The pH was measured but not controlled during fermentations. Dissolved oxygen levels were maintained at 30% saturation by adjusting the impeller(s) tip speed. Temperature, DO, and impeller tip speed were controlled automatically by the controller unit of the twin bioreactors. To minimize medium loss, the waste gas from the headspace was cooled in a reflux condenser connected to an external cooling bath (4°C) before exiting the system. Fermentations were inoculated under aseptic conditions with harvested and washed biomass from 500 mL of seed culture and were run until the initial D-glucose was depleted.

### Construction of a cexA overexpression strain

The strain overexpressing the citrate exporter gene *cexA* (*NRRL3_06530*) was constructed using *A. niger* strain CSFG_7003 (NRRL2270 ∆*pyrG* ∆*kusA*) as the host. The *cexA* gene was PCR amplified from *A. niger* genomic DNA and cloned into a plasmid containing 655 bp of the promoter and 700 bp of the terminator of the glucoamylase gene (*glaA*, *NRRL3_08300*), with *cexA* inserted between the promoter and the terminator. The constructed plasmid along with a CRISPR/Cas9 plasmid (33) containing a gRNA targeting *glaA* was co-introduced into CSFG_7003 as described previously (34). In the resulting *cexA^OE^* strain, a *cexA* allele replaces the coding region of *glaA*, and its transcription is under the control of the *glaA* promoter.

### Analytical methods

Mycelial dry cell weight (DCW) was determined from 5 mL culture aliquots as described (35). The biomass was harvested on a pre-weighed glass wool filter, washed with cold tap water, and dried at 80°C until constant weight was obtained.

The concentrations of D-glucose and citric acid in the growth media (the maximal value of the latter also referred to as volumetric citric acid yield) were determined by high-pressure/performance liquid chromatography (Agilent Technologies 1260 Infinity II, USA) with an H^+^ exchange column (Bio-Rad Aminex HPX-87H^+^) at T = 55°C, using isocratic elution with 10 mM H_2_SO_4_ and refractive index detection (36).

Mn^2+^ concentrations in the culture broth were determined by inductively coupled plasma quadrupole mass spectrometry (Thermo Fisher Scientific, Bremen, Germany) equipped with Hexapole Collision Cell Technology (37).

Biomass yield coefficients (*Y*_*x*/*s*_) were determined by dividing the maximal concentration of biomass (g·L^−1^) achieved during fermentation by the initial carbon source (D-glucose) concentration (g·L^−1^). Biomass production rates (g·L^−1^·h^−1^) were calculated from the increase in DCW over the time elapsed between two consecutive samplings (i.e., sampling time points); the highest value obtained was taken to calculate the maximal specific growth rate of the culture [µ; h^−1^ (38)]. Likewise, D-glucose utilization rates (g·L^−1^·h^−1^) were calculated from the steepest decrease in residual concentrations (g·L^−1^) between two consecutive samplings. Specific molar citric acid yields (*Y*_*p*/*s*_) are the ratio between the moles of citric acid produced and the moles of D-glucose consumed after the complete depletion of the D-glucose.

### Quantitative PCR analysis

Quantitative PCR was performed as described by Reinfurt et al. (16), using the same primers. The log2-fold changes of gene expression between manganese sufficiency and manganese deficiency were calculated according to the Pfaffl method (39). The housekeeping gene *actA* was used as reference gene.

### Sampling for transcriptome analysis

Fungal cultures growing in bioreactors were sampled under aseptic conditions at 24, 48, and 72 hours of cultivation time. Furthermore, 100 mL aliquots were pressed out of the vessel, and mycelia were harvested by filtration over nylon mesh and washed with cold sterile distilled water. Excess liquid was removed by squeezing between paper sheets. Fungal biomass was placed into 15 mL Falcon tubes containing 10 mL DNA/RNA Shield (Zymo Research) solution and were quickly frozen in liquid nitrogen. The tubes were stored at −80°C.

### Nucleic acid extraction from mycelia, library construction, and Illumina sequencing

These steps were all done by the service provider (Microsynth, Switzerland) and included DNAse treatment prior to RNA extraction from mycelia for NGS applications, preparation of polyA-enriched RNA libraries, sequencing on Illumina NovaSeq, and demultiplexing and trimming of Illumina adapter residuals.

### Analysis and functional enrichment of differentially expressed genes

The *A. niger* NRRL2270 is a spontaneous derivative of ATCC1015 (NRRL3). Genome sequence comparison revealed that strains ATCC1015 and NRRL3 are near identical with fewer than 10 single-nucleotide polymorphisms identified. Since the gene models of the NRRL3 are manually curated and are considered gold standard [(40, 41, https://mycocosm.jgi.doe.gov/Aspni_NRRL3_1/Aspni_NRRL3_1%20.home.html], the structural and functional annotations of NRRL3 are used as references for this study. The RNA-seq reads were corrected for sequencing errors with Rcorrector (42), trimming low-quality sequences with Skewer (43), and removing ribosomal RNA with SortMeRNA (44). The cleaned reads were mapped to NRRL3 transcripts and counted with Salmon (45), and the read counts were analyzed for differences in transcript expression between genotypes with DESeq2 (46). We first screened the transcriptome for genes whose mean transcripts per million from triplicates was >10. The resulting gene list was screened for those genes that were either up- or downregulated by Mn^2+^ deficiency at log2-fold greater than 2 or smaller than −2 at *P* ≤ 0.05. The protein sequences of the 963 genes that fulfilled these criteria were retrieved from Mycocosm (https://mycocosm.jgi.doe.gov/Aspni_NRRL3_1/Aspni_NRRL3_1.home.html#:~:text=The%20gold-standard%20genome%20of%20Aspergillus), and their identity checked by BLASTP (https://blast.ncbi.nlm.nih.gov/Blast.cgi) and conserved domain search (https://www.ncbi.nlm.nih.gov/Structure/cdd/wrpsb.cgi) using a treshold of <E−05. Putative localization of proteins was analyzed using SignalP (for secreted proteins; http://www.cbs.dtu.dk/services/SignalP/cdd/wrpsb.cgi) and TMHMM (for prediction of transmembrane helixes in proteins; http://www.cbs.dtu.dk/services/TMHMM/). In all three methods, only hits with *P* <  0.05 were accepted.

Although the genome database of *A. niger* NRRL3 contains annotations of several specific gene groups, we verified our proteome also using the carbohydrate-active enzyme database and CAZy nomenclature (http://www.cazy.org/), and the MEROPS database for proteases (https://www.ebi.ac.uk/merops/) and the corresponding nomenclature used to specify them. Identification of PKS, NRPS, and terpenoid synthases was confirmed with Antismash (https://fungismash.secondarymetabolites.org#!/start/) and SMURF (http://www.jcvi.org/smurf). To identify secreted small cysteine-rich proteins (SSCPs), the deduced protein sequences were first filtered with Microsoft Excel for those that have a protein size less than 300 amino acids and contain ≥5% cysteine, and the detected candidates then subjected to SignalP analysis (http://www.cbs.dtu.dk/services/SignalP/). Among this subset of proteins, hydrophobins were visually identified by the presence of eight cysteines, of which C2/C3 and C6/C7 occurred as pairs. Ceratoplatanins were identified by the presence of four cysteines and blastp against the NCBI database. The remaining proteins were considered as uncharacterized SSCPs.

### Reproducibility

All presented data involving fungal cultivations are the means of three independent experiments (biological replicates: starting with liquid cultures using different spore inocula), and each primary datum is the mean of two parallel measurements within the same experiment (technical replicates). Data were analyzed and visualized with Sigmaplot software (Jandel Scientific, San Jose, CA, USA). The variability of the data was characterized by standard deviations for each procedure. Quantitative data (*n* ≥ 3) were compared using ANOVA with Holm-Sidak Test for pairwise comparisons. While probability (*P*) values were often <0.001, the criterion for significance was *P* < 0.05 in all cases.

## Data Availability

The transcriptome data and the related protocols are available at GEO (https://www.ncbi.nlm.nih.gov/geo/) under the accession number PRJNA1032880.

## SUPPLEMENTAL MATERIAL

Table S1 spectrum.01906-24-s0001.xlsx Raw data of the expression of the total *A. niger* genome.

Table S2 spectrum.01906-24-s0002.xlsx The manganese dependent *A. niger* transriptome.

Table S3 spectrum.01906-24-s0003.xlsx Manganese limitation influenced genes encoding CAZymes 1.

Table S4 spectrum.01906-24-s0004.xlsx The six secondary metabolite gene clusters downregulated by manganese deficiency.

ASM does not own the copyrights to Supplemental Material that may be linked to, or accessed through, an article. The authors have granted ASM a non-exclusive, world-wide license to publish the Supplemental Material files. Please contact the corresponding author directly for reuse.
